# Alexithymia in Adolescents with Acne: Association with Quality of Life Impairment and Stigmatization

**DOI:** 10.3390/jcm11030732

**Published:** 2022-01-29

**Authors:** Marta Szepietowska, Alicja Dąbrowska, Bernadetta Nowak, Katarzyna Skinderowicz, Bartosz Wilczyński, Piotr K. Krajewski, Alina Jankowska-Konsur

**Affiliations:** 1 Student Research Group of Experimental Dermatology, Department of Dermatology, Venereology and Allergology, Wroclaw Medical University, 50-368 Wroclaw, Poland; marta.szepietowska0703@gmail.com (M.S.); alicja.dabrowska@student.umw.edu.pl (A.D.); bernadetta.nowak@student.umw.edu.pl (B.N.); katarzyna.skinderowicz@student.umw.edu.pl (K.S.); bartosz.wilczynski@student.umw.edu.pl (B.W.); 2 Department of Dermatology, Venereology and Allergology, Wroclaw Medical University, 50-368 Wroclaw, Poland; alina.jankowska-konsur@umw.edu.pl

**Keywords:** acne, alexithymia, students, adolescents

## Abstract

Alexithymia seems to be more common among patients with skin diseases. However, studies on acne patients are very limited. We conducted this study to evaluate alexithymia in adolescents with acne. In our cross-sectional study, 730 high school students (mean age: 17.05 ± 1.18 years) were recruited. The Toronto Alexithymia Scale (TAS-20) was used to measure alexithymia, the Dermatology Life Quality Index (DLQI) was employed to study quality of life (QoL), and the 6-item Stigmatization Scale (6ISS) was used to evaluate the level of stigmatization in acne subjects. Alexithymia was found in 31% of adolescents, with similar prevalence among those with and without acne (31.3% and 30.1%, respectively). The mean scoring on the TAS-20 in patients with acne (53.1 ± 12.8 points) was not significantly different from that of the non-acne group (53.5 ± 11.9 points). However, significant correlations between TAS-20 scores and QoL assessments (r = 0.332, *p* < 0.001) as well as stigmatization level (r = 0.284, *p* < 0.001) were found. These correlations were also significant for the domains of alexithymia described as difficulty in identifying feelings (DIF) and difficulty in describing feelings (DDF), but not for externally oriented thinking (EOT). The findings clearly showed that acne does not predispose to alexithymia; however, alexithymia in acne subjects is related to impaired QoL and stigmatization.

## 1. Introduction

Acne is the most common skin problem in adolescents and young adults [1,2]. It was clearly shown that acne patients are at an increased risk for the development of psychological comorbidities [3]. Acne patients frequently present with depressive symptoms and anxiety reactions [3,4]. They were also documented to have difficulties in emotion regulation [5]. It is not surprising that the quality of life (QoL) of acne patients is markedly decreased [6,7,8]. The term alexithymia originates from the Greek language meaning words without emotions, where a indicates without, lexi denotes word, and thymos represents emotions. It was introduced to medicine by Sifneos in 1972 [9,10]. Alexithymia is considered a personality trait characterized by a decreased ability to identify and verbally express emotions, a limited imaginative capacity, and externally oriented thinking [11,12]. Alexithymia usually has a negative influence on communication between patient and doctor and is considered a negative factor contributing to the final therapeutic outcome [13]. The prevalence of alexithymia in the general population was assessed as 10–13% [14,15,16]; however, it seems to be much more common in patients suffering from various disorders [17,18,19], including dermatological disorders [16,20,21,22,23]. However, the studies on alexithymia in acne patients are limited [15,24,25].

Therefore, we conducted this study to evaluate alexithymia in adolescents suffering from acne. Special attention was given to the relationship between alexithymia and QoL as well as the relationship between alexithymia and stigmatization level.

## 2. Materials and Methods

### 2.1. Study Design and Population

We conducted this cross-sectional study in selected high schools in southwest and central Poland. The representative study population was calculated as 384 with a 95% confidence level and a margin error of 5%. The Ethics Committee of Wroclaw Medical University approved the study protocol (KB-750/2021). The project was also approved by the directors of the selected schools. Participation was fully anonymous and voluntary. We conducted this study on adult students who agreed to participate. Moreover, after obtaining written informed consent from the parents of students under the age of 18 years, those students were also offered the possibility to participate in the project. Data were collected face-to-face by one of the investigators during the school’s classes. We ran the study for two months between 10 September and 10 November 2021.

Demographic data were obtained using a specially designed questionnaire, including the age and sex of the patients. The self-reported acne was documented. Moreover, students assessed the severity of their acne by marking one of five provided standardized color photographs [26]. Based on the Investigators Global Assessment Scale [27], the following severity categories were considered: no acne (normal skin), minimal acne (almost clear skin), mild acne, moderate acne, and severe acne.

### 2.2. Questionnaires Used

#### 2.2.1. Alexithymia Assessment

The Toronto Alexithymia Scale (TAS-20) was used to assess the presence of alexithymia in the study population. The TAS-20 was developed in 1994 and has been the most frequently used instrument for the measurement of alexithymia ever since [28]. It contains 20 questions. Each question is rated on the 5-point Likert scale ranging from 1 (strongly disagree) to 5 (strongly agree). Five questions are negatively keyed. The total TAS-20 score ranges from 20 to 100 points. A score of 61 points and above indicates alexithymia. Patients scoring less than 52 points are treated as non-alexithymic and those with scores between 52 and 60 points are considered as possibly having alexithymia (intermediate alexithymic). The TAS-20 questions are grouped to evaluate three domains of alexithymia, described as: difficulty in identifying feelings (DIF), difficulty in describing feelings (DDF), and externally oriented thinking (EOT) [28]. We used the Polish language adaptation of the TAS-20 by Ścigała et al. [29] for this study.

#### 2.2.2. Quality of Life Assessment

The quality of life of students who reported a presence of acne lesions was studied using the Dermatology Life Quality Index (DLQI). The DLQI was developed by Finlay et al. [30] in 1994 and quickly became the most popular dermatology-specific instrument to evaluate QoL impairment in patients with various dermatoses, including acne. It contains 10 questions with the answers grading patients’ agreement on a 4-point scale from 0 (not at all) to 3 (very much). The maximum score of the DLQI is 30 points. A higher number of obtained points indicates a greater impairment in QoL [30]. The participants in our study used the validated Polish version of this instrument [31]. The following cut-off points of the DLQI scores were used to grade the QoL impairment: 0–1 point (no effect of all), 2–5 points (small effect), 6–10 points (moderate effect), 11–20 points (very large effect), and 21–30 points (extremely large effect) [32].

#### 2.2.3. Stigmatization Assessment

A validated Polish language version of the 6-item Stigmatization Scale (6ISS) by Evers et al. was employed to assess the level of stigmatization in acne subjects [33]. This instrument consists of 6 questions and refers to the last 2 weeks. The answers are scored from 0 points (not at all) to 3 points (always). The total score is used for final analysis. Scores range from 0 to 18 points, and a higher number of points corresponds with a greater feeling of stigmatization [34].

### 2.3. Statistical Analysis

The results were statistically analyzed using the IBM SPSS Statistics v. 26 (SPSS INC., Chicago, IL, USA) software. First, the parametric and nonparametric distribution of the data were assessed. The minimum, maximum, and mean value with standard deviation were calculated. Quantitative variables were studied using the Mann–Whitney U test and Spearman’s correlation test. For the comparison of more than 2 groups the Kruskal–Wallis one-way analysis of variance was implemented. Qualitative data were analyzed with a chi-squared test. All analyses were performed as two-sided tests with a significance level of 5%.

## 3. Results

Among 730 students studied, 547 (74.9%) reported a presence of acne lesions. Severity of acne was self-assessed as follows: 59.1% of students reported minimal acne, 31.8% reported mild acne, 7.3% reported moderate acne, and 1.8% reported severe acne.

Alexithymia was documented in 226 (31%) students. Adolescents who fulfilled the criteria for at least intermediate alexithymia constituted 56% of the study group. The prevalence of alexithymia in the acne group was 31.3% and was not significantly different from the group of students without acne (30.1%). Similarly, taking into consideration both alexithymic and intermediate alexithymic subjects, there was no significant difference between students with and without acne (55.0% and 59.0%, respectively) (Table 1).

Alexithymia appeared to be significantly more common in girls suffering from acne (37.5%) than in boys suffering from acne (20.9%). The same phenomenon was observed in adolescents without acne (36.0% and 20.8%, respectively) without any significant difference between both analyzed groups. 

Moreover, the mean TAS-20 score in patients with acne (53.1 ± 12.8 points) was not significantly different from the non-acne group (53.5 ± 11.9 points). No differences in the mean scores for the three domains of alexithymia (DIF, DDF, and EOT) were found between acne students and those without acne (Table 2).

There was no correlation between TAS-20 scores and clinical severity of acne (detailed data not shown).

**Figure 1:**
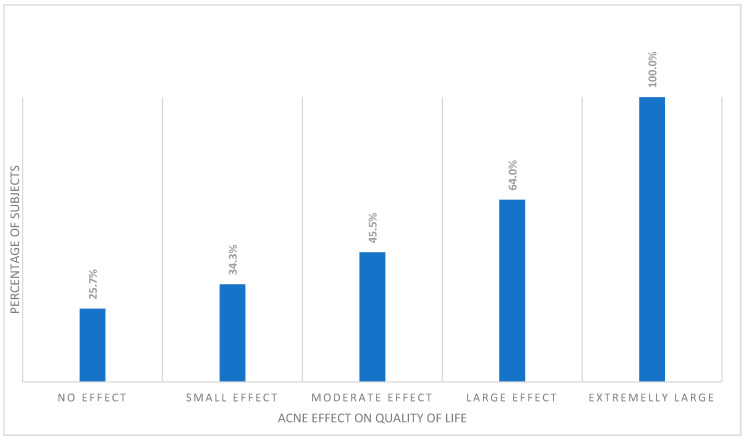
Percentage of alexithymic patients among different DLQI cut-offs.

Interestingly, despite the lack of relationship between prevalence of alexithymia and acne, among the acne subjects, alexithymia was significantly more common in those with a more decreased QoL (*p* < 0.001). All acne students with extremely large effect of their acne on QoL impairment were alexithymic. This was followed by the group of subjects experiencing large effect on QoL (64.0%), moderate effect (45.5%), and small effect (34.3%). Among acne subjects with no effect of their skin disease on QoL, only 25.7% were classified as alexithymic (Figure 1).

**Figure 2:**
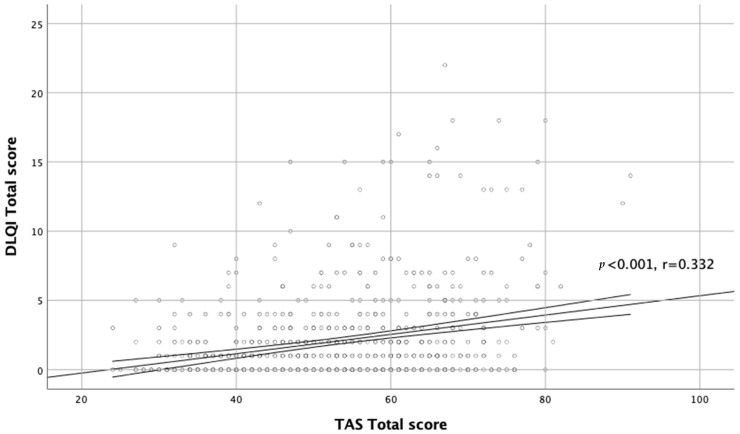
Correlation between TAS total score and DLQI total score.

Moreover, there was a significant correlation between TAS-20 scores and QoL assessment (r = 0.332, *p* < 0.001; Figure 2).

After analyzing the domains of alexithymia we documented the significant correlations between DIF and DLQI (r = 0.316, *p* < 0.001) and between DDF and QoL (r = 0.207, *p* < 0.001). EOT did not correlate with QoL impairment in our cohort of acne subjects. The same relationships were noted in both sex groups with acne (Table 3).

**Figure 3:**
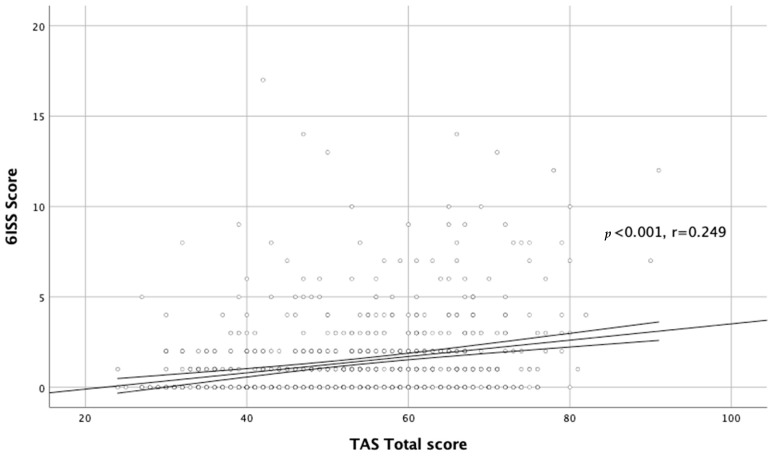
Correlation between TAS total score and DLQI total score.

We also found a significant relationship between alexithymia and stigmatization level in the whole acne group (r = 0.284, *p* < 0.001; Figure 3).

Similar to QoL, DIF and DDF correlated with stigmatization (r = 0.294, *p* < 0.001 and r = 0.314, *p* < 0.001, respectively) and there was no correlation between EOT and stigmatization level. Similar relationships between total score of alexithymia as well as scores in all three alexithymia domains were found in girls. In male subjects, there was no significant correlation between total score of alexithymia and stigmatization level; however, DIF and DDF scores correlated with the stigmatization score as observed in the female group (Table 3). 

## 4. Discussion

Acne is a chronic inflammatory disorder of the pilosebaceous unit predominantly affecting adolescents. It was suggested that up to 90% of teenagers and young adults may suffer from acne of varying clinical severity [35]. The lesions are mostly localized on the face and are visible to others [2]. Acne is frequently considered a self-limiting condition, and it seems that not enough attention is paid to appropriate acne management in the general population [35]. Although acne is not a life-threatening disease, it is responsible for important psychological burdens [3,35]. The available literature on the impact of acne on psychosocial well-being is vast. Acne is associated with higher incidence of depression and anxiety and a lowered quality of life [36,37]. It was also reported that it shapes the self-esteem and sexual satisfaction of the affected individuals [38]. Moreover, the chronic course of the disease may cause frustration and be associated with anger and mental infirmity [39].

The literature on alexithymia in acne patients is rather scarce. To the best of our knowledge, our study, based on the evaluation of 730 high school students, is the largest to date. In our study, alexithymia was found in 31.3% of acne students and in 30.1% of the non-acne group, indicating that acne is not associated with an increased prevalence of alexithymia among adolescents. Our results are in accordance with other studies. Sunay et al. [24] also used the TAS-20 to assess alexithymia in 111 acne patients aged 15 to 25 years and 78 people in a matched control group without acne lesions. They were not able to find any significant difference in the incidence of alexithymia in acne and non-acne groups. According to their results, 23.4% of acne patients and 24.4% of subjects free from acne were classified as alexithymic. Moreover, as in our study, there was no significant difference in the domains of alexithymia (DIF, DDF, and EOT) between subjects with and without acne [24]. Similarly, Dehghani et al. [15] did not reveal enhanced alexithymia levels in their acne patients; however, the number of enrolled subjects was rather low (only 30 acne patients and 30 healthy individuals studied). A cross-sectional study of 50 adolescents with acne, performed in Tunisia, demonstrated that 46% of those patients presented with symptoms of alexithymia. No control group was employed, so it was not possible to compare the prevalence of alexithymia in acne and non-acne subjects [25]. 

It is worth noting that the reported prevalence of alexithymia in both our cohort of adolescents and in the Turkish study [24] was much higher than previously estimated for the general population, for which it was around 10% to 13% [40]. The difference might be due to differences in the populations studied, especially the different age groups. Moreover, our study was performed during the COVID-19 pandemic and the studied participants were exposed to an increased level of psychological stress [41,42]. It is well-known that the prevalence of alexithymia is strongly associated with the perceived stress level both in adults and adolescents [43,44,45].

Although acne does not seem to predispose to increased alexithymia, many other skin diseases have been linked with higher scores of alexithymia. Patients suffering from psoriasis [15,46,47,48], atopic dermatitis [20,49], vitiligo [15,50], hidradenitis suppurativa [22,51], and alopecia areata [15,52] frequently scored significantly higher on the TAS-20 and other alexithymia scales. The results were not always identical, most probably due to different methodologies used, e.g., some investigators did not show the association between psoriasis and alexithymia [53,54]. In our cohort of acne patients there was no relationship between alexithymia scores and severity of acne. The same was observed by Sunay et al. [24] in their acne group. There was also no correlation between alexithymia scores and clinical severity of psoriasis [46,55], atopic dermatitis [20], or hidradenitis suppurativa [22,56]; however, some authors found such relationship in atopic dermatitis patients [49].

Despite the fact that we found no significant differences in the frequency and intensity of alexithymia between acne and non-acne subject groups, we were able to demonstrate a correlation between alexithymia and QoL impairment in patients suffering from acne. A significant correlation between TAS-20 scores and DLQI scores was documented. Similar relationships in other skin diseases, such as psoriasis [57], atopic dermatitis [20], and hidradenitis suppurativa [56] have previously been reported. Moreover, such associations between alexithymia and QoL have been found in non-dermatological diseases, including Parkinson’s disease [58,59]. We also showed that in acne subjects, alexithymia was significantly associated with a higher stigmatization level. To the best of our knowledge, this is the first report demonstrating the relationship between alexithymia and stigmatization level. We were not able to find any data concerning this issue in skin diseases or in non-dermatological conditions.

We are aware of the limitations of our study. We used the TAS-20 scoring system in our study population as in an adult population. Though a few studies have demonstrated that the TAS-20 and the EOT domain are not ideal for assessing alexithymia in this population, there are no well-founded data on different scorings [60]. Moreover, in our results the EOT domain did not correlate with DLQI nor 6ISS. It is also important to underline that patients with alexithymia might be less prone to participate in our study. Nevertheless, out of 738 students asked to participate only 8 declined. Therefore, we think that the response rate of 98.9% is sufficient for adequately reflecting this population.

## 5. Conclusions

In conclusion, our study showed that acne does not predispose to alexithymia; however, in subjects with acne, alexithymia is significantly associated with impaired QoL and increased stigmatization level.

## Figures and Tables

**Table 1:** Alexithymia in pupils with and without acne.

Alexithymia, Number of Subject (%)	Whole Group (n = 730)	Acne Group (n = 476)	Non-Acne Group (n = 254)	*p*
Alexithymia	226 (31.0)	144 (30.3)	82 (32.3)	NS
Intermediate alexithymia	183 (25.1)	116 (24.4)	67 (26.4)	NS
No alexithymia	321 (44.0)	216 (45.4)	105 (41.3)	NS

n—number of subjects; NS—not significant.

**Table 2:** Alexithymia in pupils with and without acne.

Toronto Alexithymia Scale (Points)	Acne Group (n = 476)	Non-Acne Group (n = 254)	*p*
Total score	53.1 ± 12.8	53.5 ± 11.9	NS
DIF subscale	19.7 ± 7.1	19.7 ± 6.7	NS
DDF subscale	15.2 ± 5.1	15.4 ± 4.8	NS
EOT subscale	19.1 ± 4.4	18.4 ± 4.4	NS

TAS-20—Toronto Alexithymia Scale; DIF—difficulty in identifying feelings; DDF—difficulty in describing feelings; EOT—externally oriented thinking; n—number of participants; NS—not significant.

**Table 3:** Correlations between alexithymia and quality of life impairment as well as stigmatization in pupils with acne.

Toronto Alexithymia Scale (TAS-20)	DLQI Total Score	DLQI Score Girls	DLQI Score Boys	6ISS Total Score	6ISS Girls	6ISS Boys
Total score	*p* < 0.001 r = 0.332	*p* < 0.001 r = 0.283	*p* < 0.001 r = 0.332	*p* < 0.001 r = 0.249	*p* < 0.001 r = 0.286	NS
DIF subscale	*p* < 0.001 r = 0.316	*p* < 0.001 r = 0.346	*p* < 0.001 r = 0.294	*p* < 0.001 r = 0.314	*p* < 0.001 r = 0.308	*p* = 0.007 r = 0.186
DDF subscale	*p* < 0.001 r = 0.207	*p* < 0.001 r = 0.211	*p* < 0.001 r = 0.267	*p* < 0.001 r = 0.277	*p* < 0.001 r = 0.288	*p* = 0.003 r = 0.201
EOT subscale	NS	NS	NS	NS	NS	NS

TAS-20—Toronto Alexithymia Scale; DLQI—Dermatology Life Quality Index; 6ISS—6-Item Stigmatization Scale; DIF—difficulty in identifying feelings; DDF—difficulty in describing feelings; EOT—externally oriented thinking; n—number of participants; NS—not significant.

## Data Availability

Data are not available.
